# Biological Activity and Thrombogenic Properties of Oxide Nanotubes on the Ti-13Nb-13Zr Biomedical Alloy

**DOI:** 10.3390/jfb14070375

**Published:** 2023-07-18

**Authors:** Agnieszka Stróż, Maciej Gawlikowski, Katarzyna Balin, Patrycja Osak, Julian Kubisztal, Maciej Zubko, Joanna Maszybrocka, Karolina Dudek, Bożena Łosiewicz

**Affiliations:** 1 Institute of Materials Engineering, Faculty of Science and Technology, University of Silesia in Katowice, 75 Pułku Piechoty 1A, 41-500 Chorzów, Poland; agnieszka.stroz@us.edu.pl (A.S.); patrycja.osak@us.edu.pl (P.O.); julian.kubisztal@us.edu.pl (J.K.); joanna.maszybrocka@us.edu.pl (J.M.); 2 Faculty of Biomedical Engineering, Silesian University of Technology, 40 Roosevelt, 41-800 Zabrze, Poland; maciej.gawlikowski@polsl.pl; 3 Artificial Heart Laboratory, Professor Zbigniew Religa Foundation of Cardiac Surgery Development, 345a Wolności, 41-800 Zabrze, Poland; 4 August Chełkowski Institute of Physics, Faculty of Science and Technology, University of Silesia in Katowice, 75 Pułku Piechoty 1A, 41-500 Chorzów, Poland; katarzyn.balin@us.edu.pl; 5 Department of Physics, Faculty of Science, University of Hradec Králové, Rokitanského 62, 500 03 Hradec Králové, Czech Republic; maciej.zubko@us.edu.pl; 6 Institute of Ceramics and Building Materials, Refractory Materials Center, Łukasiewicz Research Network, Toszecka 99, 44-100 Gliwice, Poland; karolina.dudek@icimb.lukasiewicz.gov.pl

**Keywords:** anodization, biological activity, biomaterials, drug delivery system, oxide nanotubes, Ti-13Nb-13Zr alloy, athrombogenity

## Abstract

The success of implant treatment is dependent on the osseointegration of the implant. The main goal of this work was to improve the biofunctionality of the Ti-13Nb-13Zr implant alloy by the production of oxide nanotubes (ONTs) layers for better anchoring in the bone and use as an intelligent carrier in drug delivery systems. Anodization of the Ti-13Nb-13Zr alloy was carried out in 0.5% HF, 1 M (NH_4_)_2_SO_4_ + 2% NH_4_F, and 1 M ethylene glycol + 4 wt.% NH_4_F electrolytes. Physicochemical characteristics of ONTs were performed by high-resolution electron microscopy (HREM), X-ray photoelectron spectroscopy (XPS), and scanning Kelvin probe (SKP). Water contact angle studies were conducted using the sitting airdrop method. In vitro biological properties and release kinetics of ibuprofen were investigated. The results of TEM and XPS studies confirmed the formation of the single-walled ONTs of three generations on the bi-phase (α + β) Ti-13Nb-13Zr alloy. The ONTs were composed of oxides of the alloying elements. The proposed surface modification method ensured good hemolytic properties, no cytotoxity for L-929 mouse cells, good adhesion, increased surface wettability, and improved athrombogenic properties of the Ti-13Nb-13Zr alloy. Nanotubular surfaces allowed ibuprofen to be released from the polymer matrix according to the Gallagher–Corrigan model.

## 1. Introduction

Each implant introduced into a living organism is initially treated as a foreign body and causes a defensive reaction of the immune system in the form of allergy or inflammation. The material that is to be used to produce a biomedical implant should have many features that will prove high biocompatibility, called biotolerance. It should show very good corrosion resistance and no adverse impact of degradation on the surrounding body cells. Ensuring high biocompatibility has become the reason for a far-reaching reduction of the content of harmful elements in metallic biomaterials [1,2,3,4,5,6,7]. The literature discusses in detail the influence of the excess of some biocompatible elements in the human body on human organs and tissues, such as DNA damage and mutations, carcinogenicity, and metal sensitivity [8].

Currently, the most common titanium alloys for biomedical applications include the Ti-13Nb-13Zr alloy [9,10,11,12,13,14]. The superiority of the willingness to use Ti-13Nb-13Zr alloy includes the fact that there is less release of metal ions during the spontaneous passivation of Ti-13Nb-13Zr because the corrosion products of the smaller alloy elements Nb and Zr are less soluble than Al and V. In addition, the native oxide layer on the alloy surface reveals higher resistance to corrosion and provides better protection for the underlying alloy [4,13,14,15,16,17]. The combination of the three most biocompatible elements, i.e., titanium, niobium, and zirconium, that do not show toxic or carcinogenic reactions with tissue and cells, allowed this alloy to be classified as the most promising material for bone implants [8,18].

Titanium and its alloys are used as implants for the osteosynthesis of the limbs and the skull and face, dental implants, some elements for contact joints, implants for cardiac surgery, implants for laryngology, elements for reconstruction, knee and hip endoprosthesis and bone implants. Implants are artificial bodies implanted in the body to recreate the natural function or aesthetics of the damaged organ. Implants replacing hard tissues are most commonly used [1,2,7,8]. The success of implant treatment is mainly dependent on the osseointegration of the implant [2,19,20,21]. It is clinically confirmed by the lack of mobility of the implant and no signs of inflammation. The process of structural and functional connection between the tissue and the implant is influenced by many factors, like the technique of inserting the implant, its stabilization, bone quality, physical properties of the bioimplant, and its shape and surface [2,5,22,23]. The first condition necessary for the correct course of the osseointegration process is to ensure the primary stabilization of the implant. The stabilization is influenced by the surface structure, bone density at the site of implementation, and the shape and size of the biomaterial. It has been proven that the denser the bone in which the implant is implanted then, the better the primary stabilization. The surface of the bone and implant materials play an important role in osteoblast adhesion and bone growth. Many factors affect the quality of the connection between bone and implant, including the chemical composition as well as the morphology of the implant surface [2,24]. Appropriate development of the implant surface seems to be an important factor for determining the level of interaction between tissues and biomaterials; therefore, the modification methods used are aimed at obtaining the appropriate surface roughness, both at the micro- and nano-scale. In vivo studies demonstrate smooth predisposing surfaces for fibrous tissue formation and rough surfaces at a microscale lead to more favorable bone formation. The rough surface, due to the presence of micro-latches, affects better anchoring but also the distribution of pressure on the surrounding environment between the implant and human tissue. Moreover, the mentioned implants, compared to those with a smooth surface, show better contact with the bone, provide greater mechanical support, and result in faster integration of osseointegration compared to implants with a smooth surface [2,25,26].

Nano-engineered surfaces have the unique ability to directly interact with cells on the overall biological response of implanted biomaterial. Therefore, various nanotechnology-based techniques have been developed to produce nano-scale surfaces on existing biocompatible implant materials. Porous nanostructured titanium alloy oxide layers in the form of nanotubes make them more suitable surgical materials for implantation. Based on numerous studies, it has been proven that implants covered with a layer of oxide nanostructures have the ability to exhibit antibody properties and thus inhibit microbial infections. Titanium nanotube materials can provide a continuous drug delivery system of the pharmacological agent to the specific place in the body where it is needed, ensuring adequate treatment over a longer period [4,27,28,29,30,31].

Anodizing is one of the techniques for modifying the surface of titanium and titanium-based biomaterials [32,33,34,35,36,37]. Electrochemical oxidation of biomedical Ti-13Nb-13Zr alloy in aqueous solutions containing fluoride ions allows obtaining an anodic nanotubes layer protecting against corrosion and the passage of metal ions, as well as improves the bioactivity of the implant [13,32,33]. The electrolyte selection for electrochemical oxidation allows assigning the obtained layers of nanotubes to one of several generations [13,34,35,36,37].

Due to our interest in the modification of the surface of the newest group of titanium alloys, this research was undertaken to study the effect of anodizing conditions on various biological properties of three generations of oxide nanotubes (ONTs) obtained on the Ti-13Nb-13Zr alloy surface. The aim of the research was to produce first-generation (1G), second-generation (2G), and third-generation (3G) ONTs and investigate their influence on biological properties such as cytotoxity and adhesion, thrombogenic, hemocompatibility, wettability and how they act as drug delivery system.

## 2. Materials and Methods

### 2.1. Preparation of Research Material

The investigated disc-shape samples of the Ti-13Nb-13Zr (wt.%) alloy were prepared by cutting the rod with a diameter of 0.9 mm. Samples after grinding with abrasive paper with a gradation of 1200 and 2500# were polished using the OP-S suspension and next washed in an ultrasonic washer in ultrapure water (Milli-Q, 18.2 MΩ cm^2^) for 20 min. Electrochemical production of 1G ONTs was carried out at room temperature at the voltage 20 V for 2 h in 0.5% HF solution. Moreover, 2G ONTs were obtained at room temperature in 1M (NH_4_)_2_SO_4_ solution with 2 wt.% of NH_4_F addition at the voltage 20 V for 120 min. Also, 3G ONTs were produced at room temperature in 1M C_2_H_6_O_2_ solution with 4 wt.% content of NH_4_F. The time of anodizing was 80 min at the voltage of 50 V. Hydrofluoric acid (48% HF), ammonium sulfate (≥98.5% trace metals basis), ethylene glycol (anhydrous, 99.8%), and ammonium fluoride (≥99.99% trace metals basis) were used (Avantor Performance Materials Poland S.A., Gliwice, Poland). Anodizing was conducted using a Kikusui PWR800H Regulated DC Power Supply (Kikusui Electronics Corporation, Yokohama, Japan).

### 2.2. Material Characterization

The structure of formed ONTs layers was examined using high-resolution electron microscopy (HREM) technique using a JEOL JEM-3010 Transmission Electron Microscope (TEM, JEOL Ltd., Tokyo, Japan) operating at 30 kV equipped with 2k × 2k Orius TM 833 SC200D Gatan CCD camera. To produce TEM samples, the anodized alloy under investigation was suspended in isopropanol and sonicated for 60 min in an ultrasonic bath. The resulting liquid was dropped on standardized Cu grid with an amorphous carbon film, and after evaporation under normal conditions, samples were studied using TEM. The bright field images were recorded. Chemical states of the Ti-13Nb-13Zr alloy surface in the initial state and after anodizing were studied using X-ray photoelectron spectroscopy (XPS) by means of a Prevac photoelectron spectrometer with a VG SCIENTA R3000 hemispherical analyzer (Pleasanton, CA, USA). Monochromatic X-rays, characterized by energy of 1486.74 eV and power of 400 W, were applied.

### 2.3. Scanning Kelvin Probe Measurements

Contact potential difference (*CPD*) maps of the Ti-13Nb-13Zr alloy surface covered with ONTs layers were registered using SEW-PAR Model 370 device (Princeton Applied Research, Oak Ridge, TN, USA). A tungsten Kelvin probe was used (KP, ø150 µm, Princeton Applied Research, Oak Ridge, TN, USA). The surface area of 1 × 1 mm^2^ was scanned. The sample was placed at a distance of ca. 80 µm from the micro-probe. Statistical analysis of the *CPD* maps allowed us to determine histograms as well as the height and spatial parameters that quantitatively characterized *CPD* magnitudes and their distribution on the surface of tested material. The arithmetic average (*CPD*_av_), the root mean square deviation (*CPD*_rms_), the skewness (*CPD*_sk_), the excess kurtosis (*CPD*_ku_), and the autocorrelation length (*CPD*_al_) parameters were determined.

### 2.4. Wettability Measurements

Water contact angle measurements were carried out using an OCA 15EC goniometer (Future Digital Scientific Corp., Westbury, NY, USA) with an accuracy of ±0.01° by the sitting airdrop method. Moreover, 10 images of water drops with a volume of about 5 µL placed on the tested surface were recorded within 10 s. On the basis of the received images, the mean values of the contact angle (Ѳ) were determined. The average of five measurements conducted in different parts of the tested surface was taken as the final values of the Ѳ.

### 2.5. In Vitro Hemocompatibility Test

Hemocompatibility of the ONTs layers on the Ti-13Nb-13Zr alloy substrate was investigated in accordance with the ASTM F756-17 [38]. Healthy human volunteers were donors of blood (Regional Blood Donation and Blood Treatment Center, Katowice, Poland). Based on the International Committee for Standardization in Hematology (ICSH), the hemoglobin (Hb) calibration curve meeting the requirements was prepared. Phosphate buffered saline (PBS) was used for preparation of dilutions to the Hb concentration of 10(1) g L^−1^. Specimens with the determined surface area were applied at a ratio of 3 cm^2^ surface area to 1 mL of test blood solution. In case of samples with produced ONTs layers, 0.57(02) ml human blood was applied. Exposition of the final samples in human blood lasted for 4 h at 37(1) °C. After incubation for 4 h, the fluid was transferred to the test tubes and centrifuged at 3000 rpm for 15 min in a standard clinical centrifuge. The supernatant was removed, being careful not to disturb any button of erythrocytes in the test tube. Then, the solution absorbance was investigated using a spectrophotometer at a wavelength of 540 nm. Calculation of % hemolysis was based on the following Formula (1):

(1) $$\%\;hemolysis=\frac{concentration\;of\;hemoglobin\;released\;in\;supernatant\cdot 100\%}{total\;hemoglobin\;concentration\;in\;tube}.$$

### 2.6. Cell Culture and Cytotoxicity Assays

Cytotoxicity tests were performed in accordance with ISO 10993-5:2009 [39]. Mammalian cell culture monolayer consisting of L-929 mouse Fibroblast cells (Sigma-Aldrich, St. Louis, MO, USA, Lot: 10i019) was used. Extraction of the test material was performed by incubating the material with MEM supplemented with 10% FBS, Penicillin/Streptomycin, and GlutaMAX at 37(1) °C (humidified) in 5(1)% CO_2_ for 24(2) h. Quadruplicate monolayers of L-929 mouse fibroblast (passage no.: 15) cells were dose with 1×, 2×, 3× and 4× dilutions of the extract and incubated at 37(1) °C in the presence of 5(1) % CO_2_ for 24(1) h. Following the incubation, 50 μL of the MTT solution, prepared just before use, was dispended in each well and incubated for 120(15) min at 37(1) °C (humidified) in 5(1) % CO_2_. Following the incubation, the MTT solution was replaced with 100 μL isopropanol and incubated for 10 min at 37(1) °C (humidified) in presence of 5(1) % CO_2_. The percent viability for the test article and control article were determined from the blanks. Reduction of the number of living cells caused a decrease in the metabolic activity in the sample. That decrease was directly connected with the amount of blue-violet formazan, whose presence was confirmed by the optical density at 570 nm with differential filter of 650 nm.

Negative Control was HDPE (Granulat G. Motloch, LOT: C7260) extract with MEM supplemented with 10% FBS, Penicillin/Streptomycin, and GlutaMAX. Positive Control was MEM supplemented with 10% FBS, Penicillin/Streptomycin, GlutaMAX, and 30% DMSO. Blank Control was MEM supplemented with 10% FBS, Penicillin/Streptomycin, and GlutaMAX.

### 2.7. Qualitative In Vitro Cell Adhesion Assay

Duplicated test articles, with the indicated side up, were placed individually in a well of a six-well plate. The L929 mouse Fibroblast cells (Sigma-Aldrich, St. Louis, MO, USA, Lot: 10i019, passage no.:8) were harvested using trypsin and counted. To each well, 80,000 cells were added with enough MEM (supplemented with 10% FBS, Penicillin/Streptomycin, and GlutaMAX) to cover the discs. The plates were incubated at 37(1) °C (humidified) in 5(1) % CO_2_ for 24(2) h. After the incubation period, the cells were fixed in 10% PFA for 15 min, followed by the Hematoxylin and Eosin staining. PFA was replaced with Hematoxylin and incubated at room temperature for 5 min. Each well was washed 3 times with tap water. Then, Eosin was added, and plates were incubated for 5 min at room temperature, followed by 3 washes with tap water. The stained cells were visualized using Delta Optical stereomicroscope SZ-630T. The photos were captured using ScopeImage 9 software and HDCE-X5 camera.

### 2.8. Thrombogenity Test

#### 2.8.1. Blood Donation and Platelet-Reach Plasma Preparation

Blood was collected for the anticoagulant CPDA-1 from healthy human volunteers after making sure that they did not take any anticoagulants, including acetylsalicylic acid, clopidogrel, or warfarin/acenocumarol over 14 days. Storing the collected blood lasted up to 24 h at 2–7 °C. Blood count (hematology automaton BC 2800 VET, Mindray, Shenzhen, China), platelets aggregation under the adenosine diphosphate (ADP) and arachidonic acid (impedance aggregometer Multiplate, Roche, Switzerland, with ADPtest and ASPItest tests) and concentration of plasma-free hemoglobin fHB (spectrophotometer Plasma/Low Hb, Hemocue AB, Ängelholm, Sweden), were controlled before starting further measurements. Blood was qualified for further tests, provided that the blood count was normal, the platelet count was higher than 1.2 × 10^5^ L µL^−1^, the ADP test result was above 122 AUC, the ASPItest result was higher than 136 AUC, and the fHB below 0.2 g dL^−1^. Platelet-rich plasma (PRP) [40] was prepared directly before the tests by centrifugation of whole blood at 100 G for 10 min at room temperature. Plasma morphology was investigated before the use of PRP in the test. Examined samples were put into polypropylene test tubes with a flat bottom of Falcon type with a volume of 50 mL and a diameter of 30 mm. Each sample was put into a separate test tube so that the sample area, which was not subjected to final processing and polishing, was at the bottom. Test tubes were filled with 10 mL of PRP, and the studied samples were incubated with PRP for 60 min at the temperature of 37(1) °C. The test tubes used for the incubation were placed on the hematology cradle at deflection ±5° and frequency 10 cycles/minute, ensuring even coverage of the samples with platelets and their aggregates. Before the incubation ended, the examined samples were withdrawn from the test tubes and carefully washed with PBS, and next fixed in 4% buffered formalin. The material prepared in such a way was then the subject of imaging tests.

#### 2.8.2. Qualitative Thrombogenicity Scale

The analysis of thrombogenicity test was carried out on 39 specimens, including 12 specimens of the 1G ONTs, 9 specimens of the 2G ONTs, 9 specimens of the 3G ONTs, and 9 specimens of the non-anodized Ti-13Nb-13Zr alloy. Neither literature data nor standards (e.g., ISO 10993-5:2009 [39]) give quantitative method of measurement thrombogenicity of biomaterials. Therefore, in the studies described in this paper, a qualitative method was used. In order to make the measurement more reliable, the assessment was performed by two experts who used a six-level ordinal scale (Table 1) [41]. As the experts, a biologist with 25 years of experience in cell testing and a bioengineer with 20 years of experience in thrombogenicity and medical statistics were chosen.

The class 0 constituted negative control. As a reference material, polyurethane based on polyesters Bionate 55D (DSM Biomedical Inc., Geleen, The Netherlands) was chosen. This material is used in medical devices devoted to long-term contact with blood. The material samples were prepared in the form of disks of 8 mm diameter and 0.8 mm thickness by high-pressure injection. The surface roughness described by an arithmetic mean deviation of the roughness profile was *R*a = 0.16.

Class 5 constituted positive control. As a reference material, glass disks covered by collagen (Neuvitro Corporation, Vancouver, WA, USA) were chosen. Collagen is a natural fibrous protein that exists in extracellular matrix and has strong ability to activate platelets.

#### 2.8.3. Statistical Analysis Methods

Both experts evaluated the same material in six-level ordinal scale. In order to study the degree of compliance of the two experts the alpha–Krippendorf coefficient was used. The coefficient has values from 0 to 1. It is agreed that if its value is >0.80, the compliance of assessments is very good. The Kruskal–Wallis test was used for that data analysis because the number of classes was higher than 2, variable describing thrombogenicity was on ordinal scale, and the experimental model was independent. The Kruskal–Wallis test is nonparametric equivalent of variance analysis. Multiple comparison test was applied for post-hoc analysis.

### 2.9. Drug Delivery System

The Ti-13Nb-13Zr samples with ONTs layers were subjected to surface functionalization heparine–dopamine (Hep-DOPA) conjugate by mixing 40 mg mL^−1^ heparin, 19.06 mg mL^−1^ EDAC (*N*-(3-Dimethylaminopropyl)-*N*′-ethylcarbodiimide, hydrochloride) and 11.5 mg mL^−1^ NHS (*N*-Hydroxysuccinimide) and reacted with 10 mL of MES buffer pH = 4.5 for 10 min (solution 1). Then, 102.2 mg mL^−1^ dopamine was mixed with 1 mL MES buffer pH = 4.5 (solution 2). Solution 1 and solution 2 were mixed and reacted for 12 h in the dark. The Ti-13Nb-13Zr alloy with ONTs layers was placed into the mixture prepared for 12 h. The material obtained in this way was dried, then ibuprofen was immobilized on the surface of the heparinized melt by adding 10 mg mL^−1^ of ibuprofen to 10 mL of Tris-HCl at pH = 8. The reaction was conducted for 24 h at room temperature with gentle shaking. The release kinetics of ibuprofen from the nanotubular layer was investigated by immersing the sample in 15 mL PBS with pH = 7.45 at 37(1) °C for 24 h. The kinetics of the released drug was studied for 60 min, taking the solution for analysis every 10 min. Each time 1 mL of the solution was withdrawn by adding fresh solution. Using UV-VIS spectroscopy, the amount of substance released from the nanotubular oxide layer was determined. The absorbance value was measured at the wavelength λ = 257 nm, determining the absorbance value of the PBS in the first step and then the absorbed solution. Based on the following Formula (2), the percentage amount of released drug was calculated:

(2) $$\%\;drug\;\;release\;=\;\frac{actual\;\;absorbance\;-\;absorbance\;\;\;0\;h}{actual\;\;absorbance}.$$

## 3. Results and Discussion

### 3.1. FE-SEM and TEM Characterization

**Figure 1:**
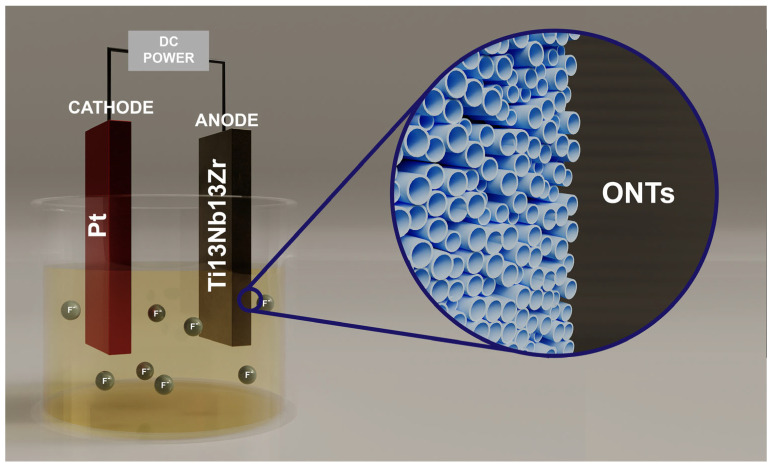
Scheme of the set-up for anodizing the Ti-13Nb-13Zr alloy in aqueous solution with fluoride ions.

The selected Ti-13Nb-13Zr alloy to obtain 1G, 2G, and 3G ONTs layers is a new type of vital titanium alloy. The addition of Nb and Zr in the amount of 13 wt.% provides appropriate properties for application in regenerative medicine. Additionally, it ensures better biocompatibility and higher corrosion resistance compared to titanium [11,12,13,14]. Anodization of the Ti-13Nb-13Zr alloy was conducted using the anode made of the tested sample and the Pt cathode at a distance of 25 mm in a face-to-face position (Figure 1).

**Figure 2:**
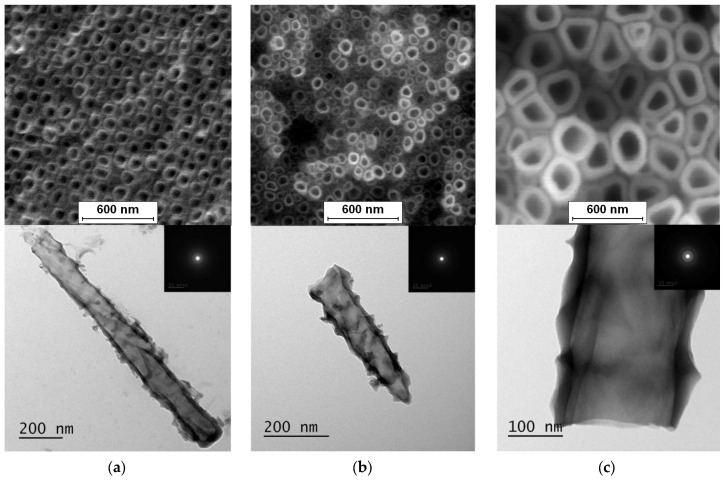
FE-SEM and corresponding below TEM images of the oxide nanotubes obtained on the Ti-13Nb-13Zr alloy: (**a**) 1G ONTs produced in 0.5% HF solution [10]; (**b**) 2G ONTs produced in 1M NH_4_(SO_4_)_2_ + 2% NH_4_F solution; (**c**) 3G ONTs produced in 1M C_2_H_6_O_2_ + 4% NH_4_F solution [13].

The obtained FE-SEM images of surface morphology for 1G, 2G, and 3G ONTs layers on the Ti-13Nb-13Zr substrate with corresponding TEM images are presented in Figure 2. From the observed regions, the selected area electron diffraction (SAED) patterns were recorded. SAED patterns (see insight in Figure 2) clearly indicate that all layers of 1G, 2G, and 3G ONTs exhibit amorphous structures. From selected areas of the FE-SEM images of three generations of ONTs layers obtained via anodization, the morphological parameters of the nanotubes were determined and summed up in Table 2.

Based on the results shown in Table 2, it can be seen that each generation of ONTs is characterized by different morphological parameters. For 1G ONTs, the smallest diameter of the nanotubes is obtained. In the case of 2G ONTs, an increase of 20% in the length of the outer diameter compared to 1G ONTs is observed. More than four and a half times larger outer diameters of 3G ONTs compared to 1G ONTs is confirmed. The longest oxide nanotubes are obtained for 3G ONTs of the order of almost 10 µm. The shortest ones are observed for 1G ONTs. The proposed anodic oxidation conditions for 2G ONTs allow to obtain nanotubes almost 4 µm long.

Based on the literature, the influence of anodizing conditions on the diameters and lengths of oxide nanotubes obtained for different generations on the Ti-13Nb-13Zr alloy can be confirmed. The influence of voltage change on the diameter of 2G ONTs on the Ti-13Nb-13Zr alloy is presented in the paper [42]. In the voltage range of 10–45 V, an increase in nanotube diameter from 55 to 250 nm can be seen. For 3G ONTs on the same substrate at the voltage of 15–35 V, the outer diameter of the nanotube increased from 104(13) to 230(30) nm [43].

### 3.2. Roughness Profile Measurements

Since the roughness of the contact surface affects the adhesion and, thus, the strength of the connection, an important element for obtaining a good adhesive connection is the proper development of the contact surface of biomaterials. For titanium implants such as orthopedic and dental, it is advisable to obtain a permanent connection between the tissue and the material; therefore, the aim is to obtain an optimum surface roughness of *R*a between 1 and 3 μm, while in the case of surgical tools and implants intended, among others, for implants prepared for contact with blood surfaces with as little roughness as possible them [43,44,45].

**Figure 3:**
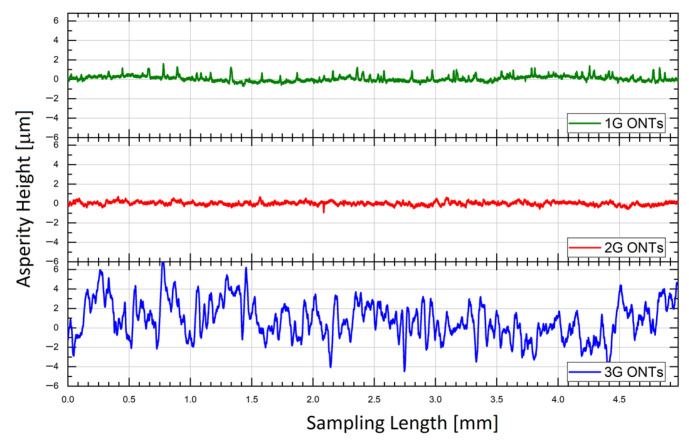
Roughness profile for the 1G, 2G, and 3G ONTs layers obtained on the Ti13-Nb-13Zr alloy via anodization.

The geometric structure of the surface (GSS) of the Ti-13Nb-13Zr alloy without and with ONTs layers of three generations was characterized by surface micro-geometry measurements in a two-dimensional (2D) system. The discussion of the influence of anodizing conditions on the GSS was based on the selected profile height parameter *R*a. Figure 3 presents the exemplary roughness profiles after alignment for the 1G, 2G, and 3G ONTs layers on the Ti13-Nb-13Zr alloy. All obtained surface profiles were symmetrical. A similar value of *R*a = 0.16(01) and *R*a = 0.13(01) parameters for the 1G and 2G ONTs layers was determined, respectively. The 3G ONTs were characterized by the highest *R*a parameter of 1.34(04).

The *R*a for the 3G ONTs increased over 8 times in comparison with the 1G ONTs and 10 times in relation to the 2G ONTs due to the differences in the morphological parameters of the obtained nanotubes on the Ti-13Nb-13Zr alloy. It can be seen that the most developed surface and the thickest layer of nanotubes were obtained for the 3G ONTs layer (Table 2). Based on the obtained results, it can be expected that the 3G ONTs layers will allow for better stability at the implant–bone interface, improved osseointegration, and reduced risk of metal ion release as products of corrosion processes and biotribological wear. In our earlier work [43], it was reported that deviations of the roughness profile from the mean line at the micro-scale were not observed for the smooth surface of the Ti-13Zr-13Nb alloy before anodizing, for which *R*a was only 0.10 µm. The positive role of surface roughness was reported in studies on stainless steel, Co-Cr alloy, titanium, Ti-6Al-4V, and Ti-6Al-7Nb alloys using human osteoblasts [46,47]. The authors studied the activity of bone tissue after implanting titanium implants with a smooth and rough surface on rabbits. Better contact of the implant with the bone and a larger volume of bone tissue was obtained for rough surfaces compared to smooth surfaces.

### 3.3. XPS Study of Chemical States

**Figure 4:**
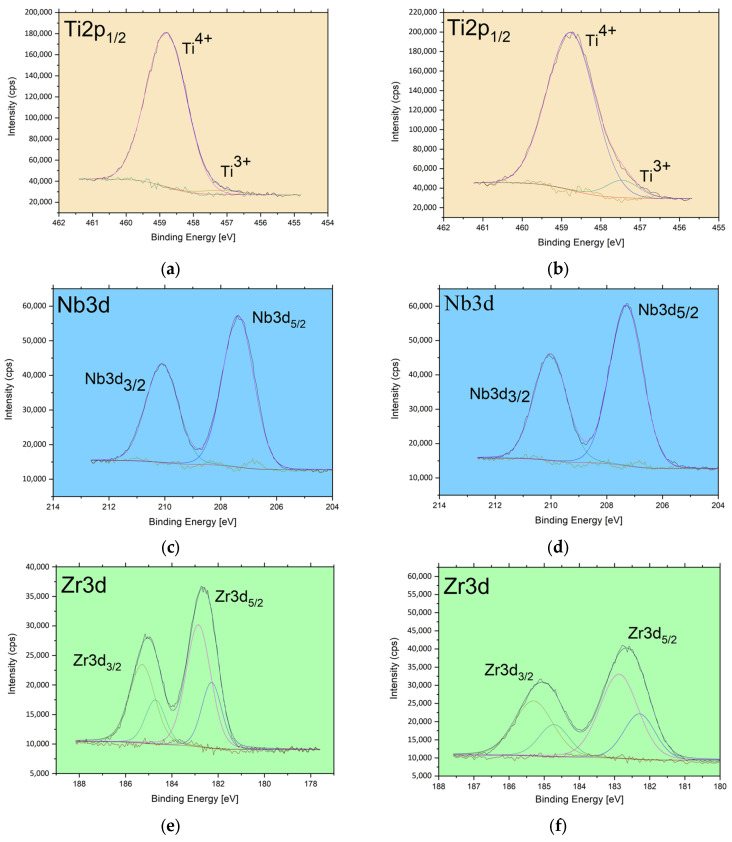

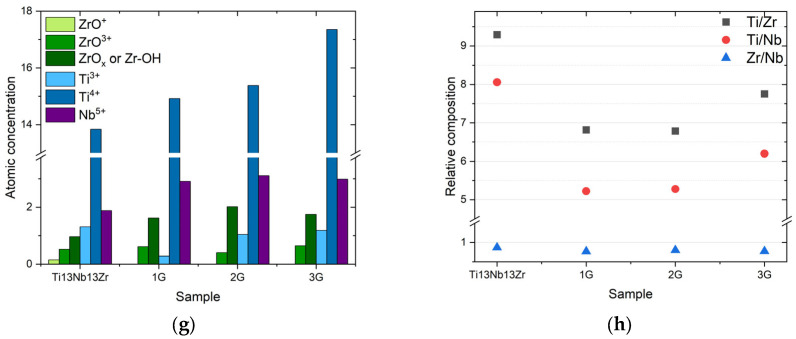
XPS high-resolution spectra for Ti2p_7/2_: (**a**) 1G ONTs, Ti2p_1/2_; (**b**) 2G ONTs, Ti2p_1/2_; (**c**) 1G ONTs, Nb3d; (**d**) 2G ONTs, Nb3d; (**e**) 1G ONTs, Zr3d; (**f**) 2G ONTs, Zr3d; (**g**) Sample-dependent atomic concentration; (**h**) Relative composition elements dependent on the sample.

The XPS studies concerned the determination of atomic concentration, and chemical states analysis of the obtained ONTs components. Detailed analysis of the shape and position of Ti2p, Nb3d, and Zr3d photoemission lines for the 1G and 2G ONTs in Figure 4 a–f was conducted and compared with the results of our previous XPS research on the Ti-13Nb-13Zr alloy reported in the earlier work [13].

The titanium in electrochemically obtained 1G and 2G ONTs occurs mainly as the TiO_2_. The binding energy of the prominent peak (Figure 4a) is located at 458.8 eV and is characteristic of the Ti^4+^ oxidation state as observed in anatase [48]. A weak contribution of oxidized titanium characteristic for trivalent titanium is visible at binding energy 457.2 eV [49]. Both chemical states were previously observed in the Ti-13Nb-13Zr alloy, for which 91.64% of titanium atoms were in the 4^+^ valence state and 8.36% in the 3^+^ [13]. The amount of the trivalent titanium in the 1G and 2G ONTs is lower than in mentioned reference sample; for the 1G ONTs, Ti^3+^ atoms account for only 1.81% of all titanium atoms, whereas for the 2G ONTs, it is only 6.35%.

The deconvolution of the lines Nb3d of the 1G and 2G ONTs indicates the presence of Nb in one chemical state—the position of the Nb3d_5/2_ at about 207.4 eV (Figure 4c,d) is characteristic of the Nb_2_O_5_ oxide [50]. The same result for niobium was observed in 3G ONTs in our previous studies [13]. Analysis of the zirconium lines in Figure 4e,f revealed some similarities between the surfaces of the 1G and 2G ONTs and previously reported electrochemically modified Ti-13Nb-13Zr. The positions of deconvoluted peaks indicate the presence of ZrO_2_ with a peak at 182.3 eV and Zr in another oxidation state with a peak at 182.9 eV or Zr(OH) [51]. The amount of Zr in a particular chemical state is different for 1G and 2G ONTs and differs even more when compared with the previously analyzed 3G ONTs sample [13].

The results of the contribution of the particular chemical state for the electrochemically obtained 1G, 2G, G3 ONTs layers, and reference alloy are summarized in Figure 4g. The data points represent the calculated atomic concentration for each atom in each chemical state. The general observation suggests that Nb and Zr bonded with oxygen or OH group in anodic ONTs layers are relatively stable—their amount does not depend on the applied procedure of electrochemical modification. In the case of Ti, applied anodization generally influences the amount of titanium but also titanium chemical states. This is worth noting that applied procedures for electrochemical oxidation of Ti-13Nb-13Zr in all three generations lead to changes in the relative ratios of the elements under consideration (Figure 4h). Those changes are related to titanium as only the relative ratios of Ti/Zr and Ti/Nb are changed when compared with the reference sample. Based on the 1G and 2G ONTs layers studies, it can also be observed that even if the relative amount of sample constituents are almost identical, as observed for the 1G and 3G [13] ONTs layers, their chemical composition is different.

### 3.4. Electronic Properties

**Figure 5:**
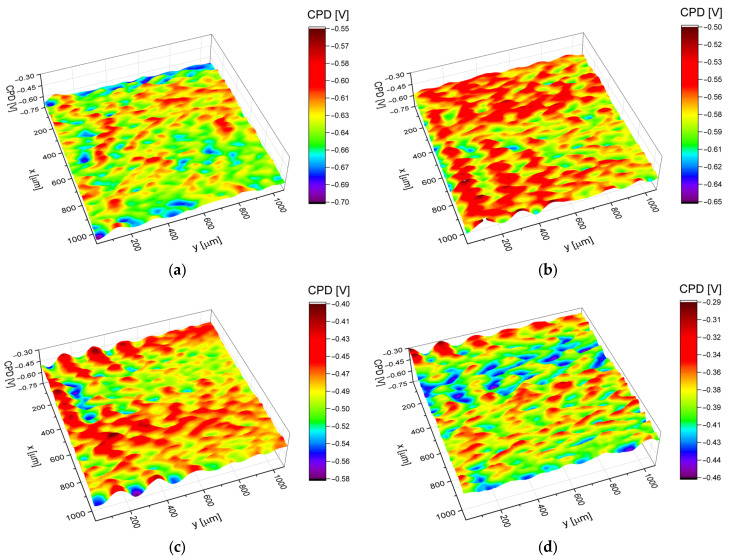
*CPD* maps determined for (**a**) Ti-13Nb-13Zr alloy; (**b**) 1G ONTs; (**c**) 2G ONTs; (**d**) 3G ONTs.

Statistical analysis of the contact potential difference maps in Figure 5 allowed us to determine the arithmetic average (*CPD*_av_), the root mean square deviation (*CPD*_rms_), the skewness (*CPD*_sk_), the excess kurtosis (*CPD*_ku_), and the autocorrelation length (*CPD*_al_), i.e., parameters that quantitatively describe electronic properties of the Ti-13Nb-13Zr alloy surface covered with ONTs. The values of the above parameters are presented in Table 3.

It was found that the substrate surface covered with ONTs is characterized by the higher value of the *CPD*_av_ in comparison with the biomedical Ti-13Nb-13Zr alloy, regardless of the solution composition from which nanotubes were obtained. Moreover, it was stated that the 3G ONTs are characterized by the highest *CPD*_av_ value (ca. −387 mV_KP_) among all the investigated biomaterials. This result may be related to the different chemical compositions of solutions and hence different concentrations of fluorine ions in electrolytes (Table 2), from 0.5% F^−^ for the 1G ONTs, 2% for the 2G ONTs, and 4% for the 3G ONTs.

The root mean square deviation (*CPD*_rms_) determines the deviation of *CPD* heights in relation to the arithmetic average, whereas autocorrelation length (*CPD*_al_) characterizes the spatial distribution of *CPD* heights on the material surface. In other words, *CPD*_al_ is a measure of the distance over the surface by which one would find a surface feature (*CPD* in this case) that is statistically different from the one that can be found in the original location. The *CPD*_rms_ parameters obtained for the investigated surfaces indicate increasing heights of *CPD* in the order Ti-13Nb-13Zr, 1G ONTs, 2G ONTs, and 3G ONTs. Simultaneously values of the *CPD*_al_ parameter show that the correlation length is the highest for the Ti-13Nb-13Zr substrate and decreases for surfaces covered with the nanotubes. Generally, both parameters i.e., *CPD*_rms_ and *CPD*_al_ indicate that the Ti-13Nb-13Zr substrate is characterized by the most uniform surface of all the samples tested and also that the production of nanotubes on the Ti-13Nb-13Zr surface increases its electrical heterogeneity. The increase in *CPD*_rms_ and *CPD*_al_ determined for the surfaces covered with ONTs can be explained by increasing the dimensions of outer diameters of the nanotubes from 87(10) nm for the 1G ONTs, 103(10) nm for the 2G ONTs, and 342(34) nm for the 3G ONTs. Skewness (*CPD*_sk_) and excess kurtosis (*CPD*_ku_) describe the shape of the *CPD* distribution and give additional information on the *CPD* peaks/valleys profile. It was found that for all investigated samples *CPD*_sk_ and *CPD*_ku_ parameters change in the range ±0.3 (see Table 3); thus, one can state that for each material tested, the *CPD* distribution follows a Gaussian (normal) distribution.

**Figure 6:**
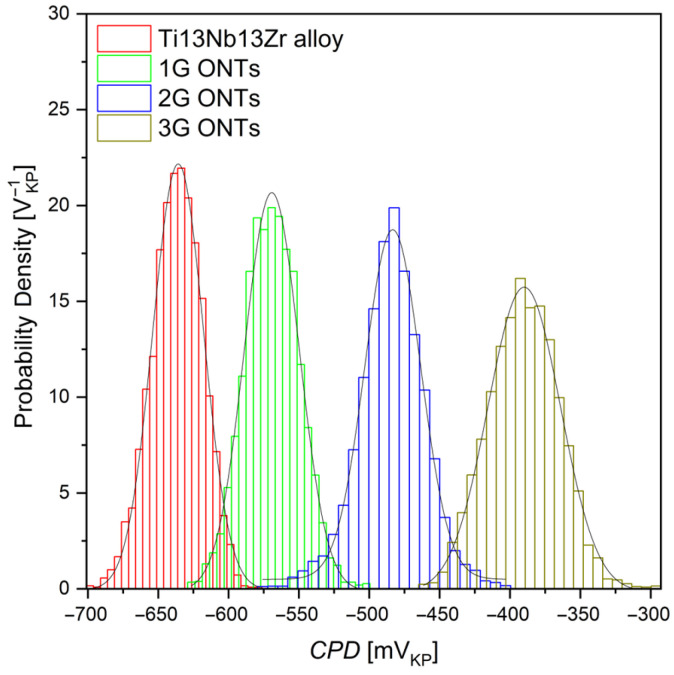
*CPD* histograms determined for the Ti-13Nb-13Zr alloy and for the 1G, 2G, and 3G ONTs; solid lines—fit of the Gaussian function.

Histograms of the *CPD* values with fitted Gaussian distributions are shown in Figure 6. The comprehensive description of the histogram preparation and the determination of the parameters can be found elsewhere [52,53,54].

Approximation of the histograms using the Gaussian function allows determining the arithmetic average *CPD*_av_ and the standard deviation, which in this case corresponds to the *CPD*_rms_ parameter. Values of *CPD*_sk_ and *CPD*_ku_ also indicate that the *CPD* heights are symmetrically distributed around the average as well as that the investigated surfaces do not show inordinately high peaks/deep valleys. Generally, one can state that the entire surface of the Ti-13Nb-13Zr alloy is evenly covered with ONTs irrespective of the different production processes (solution composition, anodization parameters).

### 3.5. Surface Wettability

**Figure 7:**
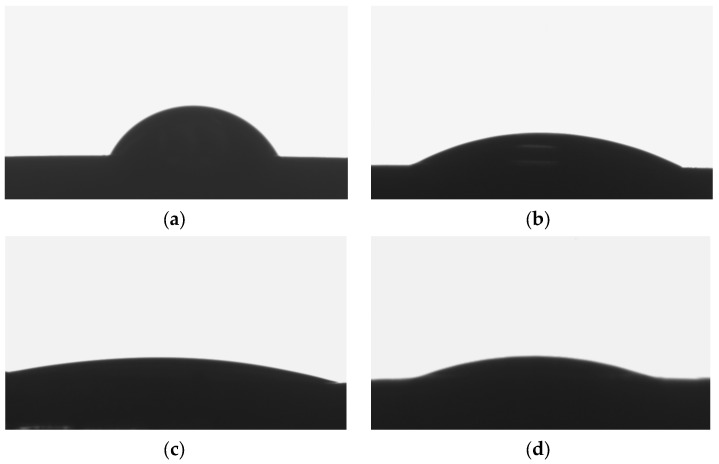
Image of a water drop on the surface of the Ti-13Nb-13Zr alloy: (**a**) Non-anodized; (**b**) Anodized in 0.5% HF; (**c**) Anodized in 1 M (NH_4_)_2_SO_4_ + 2% NH_4_F; (**d**) Anodized in 1 M C_2_H_6_O_2_ + 4% NH_4_F.

The nature of the interaction of the biomaterial with the tissue, related to each of the key properties of the surface layer mentioned above, is also determined by surface wettability. It determines protein adsorption, blood coagulation, and biological response. Most of the commonly used biomaterials are hydrophobic and have a high affinity for many proteins. Immediately after implantation, the biomaterial is covered with a layer of proteins. These are mainly albumin, fibrinogen, fibronectin, immunoglobulins, and von Willebrand factors. In shaping the surface properties of implants, wettability should also be taken into account, which is important for the osetoinduction process preceding osseointegration and affects the absorption of molecules favoring the adhesion of fibroblasts and/or bacteria [55,56]. Hydrophilic surfaces have better biological activity in contact with body fluids and, thus, provide better osseointegration [57]. The surface wettability tests of the produced ONTs layers showed that the contact angle measured for the Ti-13Nb-13Zr alloy without and with vertically oriented 1G, 2G, and 3G ONTs took the following Ѳ values in sequence: 62.9(9) °, 25.3(5) °, 11.9(1) °, and 14.7(2) °, respectively (Figure 7).

The tests performed confirmed the favorable hydrophilic nature of the produced ONTs layers. Investigation of the contact angle of the individual sample surface showed that they were hydrophilic in nature both before and after anodization (Figure 7a–d). A reduction in Ѳ value was observed for the samples after anodization, which indicates an increase in hydrophilicity. The most advantageous hydrophilic properties showed the 2G ONTs, then 3G ONTs, 1G ONTs, and the smallest Ti-13Nb-13Zr alloy. The obtained wettability results of nanotubular surfaces on titanium are similar [58]. Using anodization as a method of electrochemical modification enhances the surface wettability of titanium and its alloys.

### 3.6. In Vitro Hemocompatibility Study

**Figure 8:**
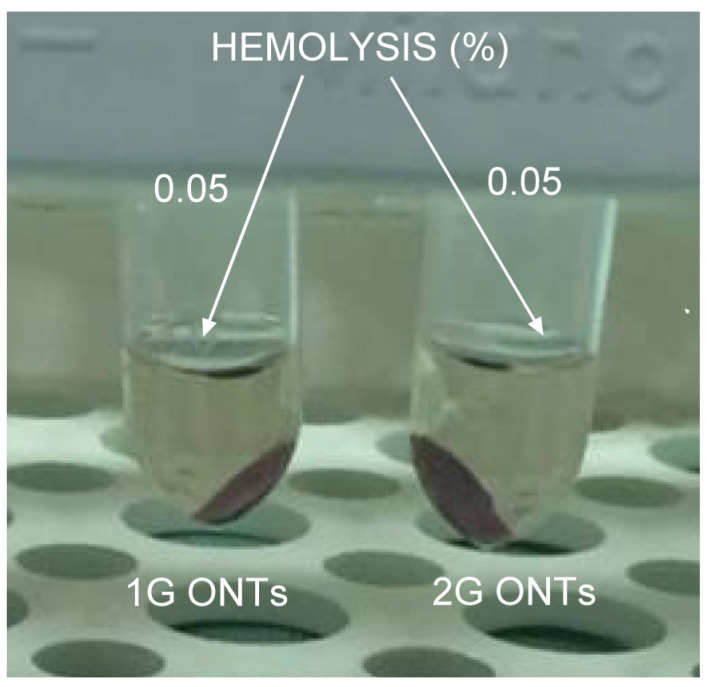
Anti-hemolytic activity of the 1G and 2G ONTs on the Ti-13Nb-13Zr alloy.

Currently, the most popular method of determining hemocompatibility is the hemolysis test. The process of hemolysis is based on the transition of hemoglobin into the cytoplasma due to the rupture of erythrocytes. According to the ASTM F756-17 standard [38], the biocompatibility of the 1G and 2G ONTs examinations were performed (Figure 8).

The value of the hemolytic index lower than 2 indicates that the biomaterial is nonhemolytic. The obtained results of in vitro hemocompatibility study shown in Figure 8 revealed the value of the hemolytic index of 0.05 for both the 1G and 2G ONTs. We found the hemolytic index for the 3G ONTs to be 0.00 [13]. The highest hemolytic index of 0.30 was reported for the non-anodized Ti-13Nb-13Zr alloy [13]. The obtained values prove that the proposed electrochemical modification ensures outstanding hemocompatibility. In the case of the 3G ONTs, it is even possible to completely eliminate hemolysis.

### 3.7. Cytotoxity and Cell Adhesion Assay

The interaction of the substrate material with a nanotubular structure with the biological environment can also be assessed during the use of cell cultures and cellular response on the surface prepared in this way. In the past, in order to evaluate the cytotoxicity of biomaterials for medicine, in vitro mammalian cell culture tests were used. At present, morphology, chemistry surface, and type of cells, especially concentration and time of exposure, determine the cytotoxicity of nanomaterials. The test article showed no cytotoxic potential to L-929 mouse fibroblast cells for the non-anodized Ti-13Nb-13Zr alloy and with the obtained 1G, 2G, and 3G ONTs layers (Table 4). In the case of oxide nanotubes produced on the vital Ti-13Nb-13Zr alloy by the anodizing method, the obtained cytotoxicity for mouse fibroblast cells is similar. The adhesion, proliferation, and migration of cells forming bone tissue are related to the diameters of nanotubes. This is confirmed by numerous studies showing that the ONTs surface is conducive to the growth of cells. It should be noted that different research groups used different cell colonies, which affected the results of the study [59,60,61].

Based on the prepared samples for cytotoxicity testing, cell adhesion to layers obtained via anodization and to the starting material was also tested. The cells adhere with different efficiency, which can be seen in Table 5. The obtained results of the adhesion test showed a different degree of adhesion depending on the tested oxide layer. For the self-passive oxide layer present on the Ti-13Nb-13Zr alloy and for the 1G ONTs, high cell adhesion was obtained. For the 2G and 3G ONTs, the medium adhesion was revealed. It can be correlated with the diameter and length of the obtained nanotubes in dependence of generation. Cell adhesion decreased with the growth and length of the ONTs.

ONTs layers are able to increase the bioactivity of the implant surface. First of all, they increase the surface roughness at the nano-scale, forming a biomimetic nanostructure, which is similar to natural rough bone tissue. Bone-forming cells (osteoblasts) have been proven in numerous studies [62,63] to adhere to a surface that is morphologically and chemically similar to natural bone tissue. The interaction of the nanotube-structured substrate material with the biological environment can also be assessed during the use of cell culture. Cell adhesion is related to the diameter of the nanotubes; research is shown in [64]. The authors showed that oxide nanotubes with a smaller diameter had a more stimulating effect on cell growth and differentiation.

### 3.8. Thrombogenicity Test

The alpha Kappendorf coefficient was 0.88, which proved very good compatibility with the thrombogenicity assessment carried out by the two experts. The relationship between the evaluations is shown in Table 6. Numbers in the table cells represent the number of samples.

**Figure 9:**
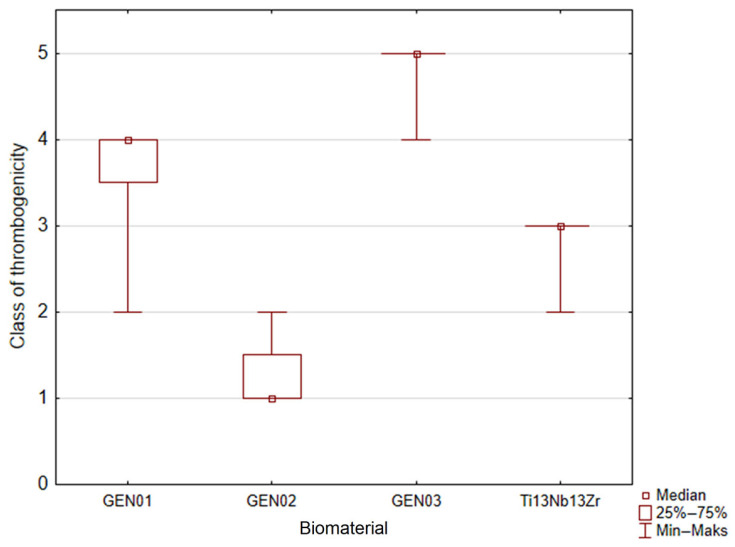
Thrombogenicity of the studied Ti-13Nb-13Zr alloy before and after formation of 1G, 2G, and 3G ONTs (in six-level ordinal scale).

The result of the Kruskal–Wallis test was significant (*p* = 0.0000), which means that at least one group differs from the rest. Results of the post-hoc test are shown in Table 7 and Figure 9.

Class 0 meant very small thrombogenicity comparable with negative control. Class 5 meant very high thrombogenicity comparable with positive control. The meaning of a given class is described in Table 1, and each description was exemplified by images of the biomaterial surfaces (Bionate 55D polymer, PEEK, Ti6Al7Nb alloy with a layer of TiN, PEEK polymer, ZrO_2_+Y_2_O_3_ ceramics, and oraz glass covered by collagen) dotted by thrombocytes and their aggregates. Usage of the interval scale limited the possibility of applying advanced statistical methods and made worse the quality of inference but, on the other hand, made carrying out a statistical proof possible.

In order to study how the expert assessments differ, the alfa Kappenrorf coefficient was used. Taken values from 0 to 1 where 0 means total discrepancy of the expert assessments and 1 means their total agreement. It is accepted in the literature data that a coefficient value larger than 0.8 means a very good agreement in the assessments. The alpha Kappendorf coefficient for the presented studies was 0.88; thus, the expert’s opinions were practically equal. Further analysis was carried out with the use of the Kruskal–Wallis tests with the post-hoc multiple comparison method.

Thrombogenicity of the 1G and 3G ONTs layers is practically the same, and those biomaterials strongly unfavorably influence the activation of coagulation factors. They were classified into classes 4 and 5, respectively. Class 5 contains samples of positive control, so the 3G ONTs biomaterial activates the coagulation system to the same degree as strongly thrombogenic collagen. Athorombogenic properties of the 1G ONTs biomaterial are only slightly better than these of the 3G ONTs, but one should remember that there are no statistically important differences between those two materials (*p* = 0.1520—see Table 4). Thrombogenicity of the 2G ONTs and Ti-13Nb-13Zr does not differ; however, these biomaterials show much better athrombogenic properties than the 1G and 3G ONTs. The 2G ONTs and Ti-13Nb-13Zr were classified into classes 1 and 3, respectively. Class 1 contained biomaterials slightly worse than the positive control (Table 1). Thus, the 2G ONTs biomaterial has a small effect on the coagulation system. The Ti-13Nb-13Zr alloy is situated in class 3; however, there is no statistical proof that it differs from the 2G ONTs (*p* = 0.4582, compared to Table 4).

### 3.9. Drug Release Kinetics

**Figure 10:**
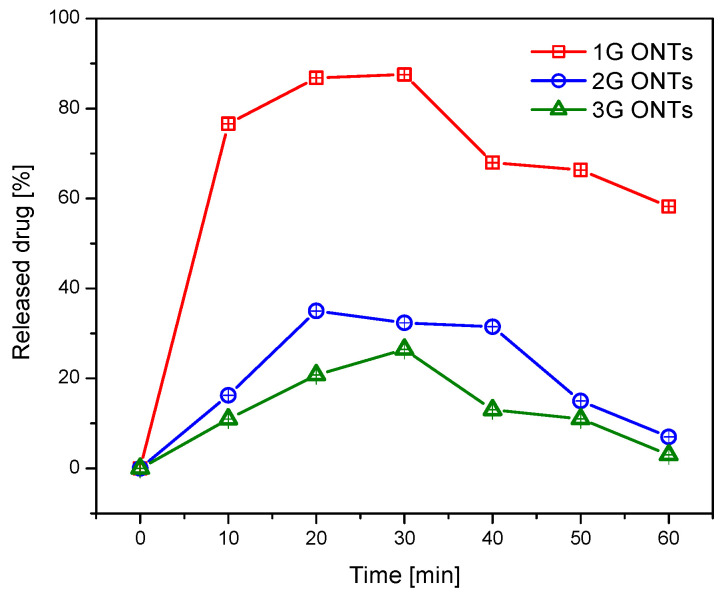
Ibuprofen release profile from the 1G, 2G, and 3G ONTs on the Ti-13Nb-13Zr alloy in function of the time.

To check the possibility of the potential use of ibuprofen-loaded Hep-DOPA/ONTs as carriers for the controlled release of the drug was compared the ibuprofen release profiles for three generations of oxide nanotubes on the Ti-13Nb-13Zr alloy obtained via anodization. The analysis of the obtained results showed a rapid increase in the released ibuprofen in the first 10 min for the 1G ONTs (Figure 10). A continuous increase in drug content is observed for up to 30 min. After this time, the ibuprofen content began to decrease, and after 60 min, the drug content dropped to 0. In the first 10 min, ibuprofen is released from 16.25% for the 2G ONTs and 10.96% for the 3G ONTs. Between 20 and 30 min, there is an increase in drug release to about 35 and 36%. From 40 min, the release of the drug decreases to less than 10% at 60 min. The high dose released from the 1G ONTs in the first minutes proves the possibility of rapid anti-inflammatory and analgesic effects by releasing the substance from the nanotubular coating, which is necessary to relieve pain in peri-implantation tissues and reduce inflammation without using oral analgesic therapy [65].

Ibuprofen (C_16_H_18_O_2_) is a non-steroidal anti-inflammatory drug, a derivative of propionic acid [66]. It has anti-inflammatory, analgesic, and antipyretic properties. It inhibits platelet aggregation. The action mechanism of ibuprofen is based on the inhibition of the activity of the COX-1 and COX-2 cyclooxygenase enzymes [66]. COX-2 enzyme activity increases rapidly in inflamed tissues. The inhibition of this enzyme by ibuprofen reduces the inflammatory process from the first moments of action. Orally administered ibuprofen has a number of side effects. Therefore, the administration of ibuprofen in the form of a drug release system reduces the risk of side effects without irritating the gastrointestinal mucosa, which is especially important in ulceration and bleeding from the stomach and duodenum [67]. Ibuprofen is a drug that cannot be used long-term, like antibiotics [67]. Therefore, the rapid release of a dose of ibuprofen in the first moment after implantation can effectively inhibit the action of the COX-2 enzyme and thus limit the development of the first inflammation in the tissues surrounding the implant. Orally administered ibuprofen disintegrates very rapidly. The disintegration time of ibuprofen in the liver is about 2 h; therefore, ibuprofen is an excellent substance for application in controlled drug release systems [68,69,70]. The drug immobilized inside the nanotubes must be soluble in water environments so that they dissolve in body fluids, in particular in blood. This condition must be met to achieve full biodistribution in the human body. The advantage of using ibuprofen is that it is 90% bound to plasma proteins [70].

In addition, the material to which the drug is applied must be biocompatible. Oxide nanotubes belong to a group of materials characterized by a developed and diversified surface, and thus a greater possibility of application of therapeutics [71]. In the case of ONTs of the 1G, 2G, and 3G with a single-walled structure, the drug is applied to the inside of the nanotube by attaching the drug to the surface with covalent amide or ester bonds [72,73]. Ibuprofen from the nanotubes is released through the so-called “burst effect”, i.e., a rapid release of the drug within the first 15 min [71,74]. The primary mechanism of drug release is the Gallagher–Corrigan model. This model describes a two-stage drug release kinetics. According to the Gallagher–Corrigan model, the first phase of drug release is its burst from the polymer Hep-DOPA matrix. The second stage is slow release, determined by matrix degradation. The rate of the drug release process in this type of drug delivery system is high in the initial period, then it decreases [71].

## 4. Conclusions

The obtained FE-SEM and TEM results confirmed the presence of the 1G, 2G, and 3G oxide nanotubes on the Ti-13Nb-13Zr alloy surface obtained via anodization in different conditions. The morphological parameters of the obtained oxide nanotubes increased with the increase in the fluorine ions content in the solution causing the increase in the surface roughness. The *R*a parameter for the 3G ONTs was over 8 times higher in comparison with the 1G ONTs and 10 times higher in relation to the 2G ONTs. XPS studies showed that these nanotubular layers of three generations were composed of TiO_2_, Nb_2_O_5_, ZrO_2_, and ZrO_x_. The electronic properties of the 1G, 2G, and 3G ONTs tested by the SKP method depended on the content of F^−^ ions. Statistical analysis of the contact potential difference maps showed that the quantitative parameters in the form of the arithmetic mean and the square root of the height irregularity increased with the increase in the content of fluorine ions in the solution from 0.5% to 4%. The 2G nanotubular oxide layers show the best hydrophilic properties. Biological assessment of hemocompatibility of the obtained ONTs revealed no hemolytic effects confirming the fulfillment of the requirements for clinical use. The best adhesion was shown by the 1G ONTs and the alloy before surface modification. The thrombogenicity of the 2G ONTs and Ti-13Nb-13Zr alloy does not differ from each other, but they have much better athrombogenic properties than the 1G and 3G ONTs. The ibuprofen release profile from the Hep-DOPA matrix from three types of oxide nanotube generation was investigated. A two-stage Gallagher–Corrigan model was selected to describe the kinetics of the ibuprofen release mechanism.

## Figures and Tables

**Table 1:** Ordinal thrombogenicity scale together with description and exemplary images of surface of the sample belonging to a given class [41].

Class	Description	Example
0	contains samples characterized by minimal thrombogenicity: separated platelets of low diverse level, no platelet aggregates	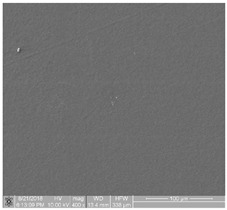
1	contains samples characterized by very low-degree thrombogenicity: a dozen or so adhered blood platelets not creating aggregates	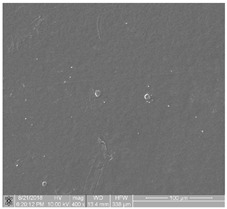
2	contains samples characterized by very low-degree thrombogenicity: several dozen of visible platelets which can be present as single, separated aggregates with a small area	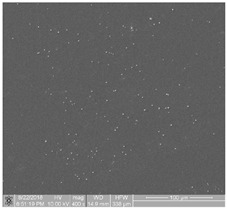
3	contains samples characterized by average-degree thrombogenicity: biological material mainly comprises aggregates larger than individual blood platelets	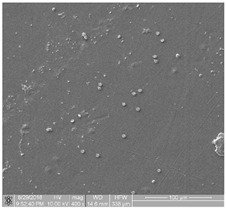
4	contains samples characterized by high-degree thrombogenicity: sample is covered with highly differentiated biological material, and the individual objects are connected with each other without possibility of separating them	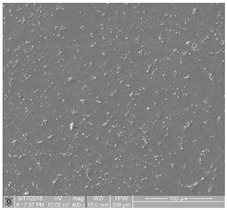
5	contains samples characterized by very high-degree thrombogenicity: platelets are highly differentiated and form numerous aggregates, which are connected with each other without possibility of separating and counting objects	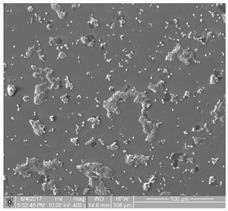

**Table 2:** Morphological parameters of 1G [10], 2G, and 3G [13] ONTs produced on the Ti-13Nb-13Zr alloy via anodization.

Electrolyte	Anodization Parameters	ONT Internal Diameter (nm)	ONT Outer Diameter (nm)	ONTs Length (μm)
0.5% HF	E = 20 V, t = 120 min	71(7)	87(10)	0.94(9)
1M (NH_4_)_2_SO_4_ +2% NH_4_F	E = 20 V, t = 120 min	61(6)	103(10)	3.9(4)
1M C_2_H_6_O_2_ +4% NH_4_F	E = 50 V, t = 80 min	169(17)	342(34)	9.7(9)

**Table 3:** Statistical parameters calculated using *CPD* maps of the Ti-13Nb-13Zr substrate and for the 1G, 2G, and 3G ONTs; *CPD*_av_ is the arithmetic average, *CPD*_rms_ is the root mean square deviation, *CPD*_al_ is the autocorrelation length, *CPD*_sk_ is the skewness, and *CPD*_ku_ is the excess kurtosis; *V*_KP_ is the voltage measured versus Kelvin probe.

Parameter	Ti-13Nb-13Zr	1G ONTs	2G ONTs	3G ONTs
*CPD*_av_ (mV_KP_)	−634.2	−566.5	−480.4	−386.7
*CPD*_rms_ (mV_KP_)	17.8	19.1	23.7	24.7
*CPD*_al_ (µm)	63.61	31.37	43.12	52.29
*CPD*_sk_	−0.20	0.06	−0.15	0.07
*CPD*_ku_	−0.09	0.05	0.28	−0.02

**Table 4:** Cell response for the Ti-13Nb-13Zr alloy before and after formation of 1G, 2G, and 3G ONTs.

Material	Percent Viability (%)	System Suitability
Positive control	0.54	No Cytotoxic Potential
Negative control	98.33	No Cytotoxic Potential
Ti-13Nb-13Zr (1×)	92.26	No Cytotoxic Potential
Ti-13Nb-13Zr (2×)	97.18	No Cytotoxic Potential
Ti-13Nb-13Zr (3×)	97.49	No Cytotoxic Potential
Ti-13Nb-13Zr (4×)	99.63	No Cytotoxic Potential
1G ONTs (1×)	100.6	No Cytotoxic Potential
1G ONTs (2×)	85.29	No Cytotoxic Potential
1G ONTs (3×)	103.8	No Cytotoxic Potential
1G ONTs (4×)	98.67	No Cytotoxic Potential
2G ONTs (1×)	87.91	No Cytotoxic Potential
2G ONTs (2×)	99.59	No Cytotoxic Potential
2G ONTs (3×)	106.3	No Cytotoxic Potential
2G ONTs (4×)	106.2	No Cytotoxic Potential
3G ONTs (1×)	88.45	No Cytotoxic Potential
3G ONTs (2×)	89.97	No Cytotoxic Potential
3G ONTs (3×)	95.02	No Cytotoxic Potential
3G ONTs (4×)	96.63	No Cytotoxic Potential

**Table 5:** Estimated cell adherence to the Ti-13Nb-13Zr alloy before and after formation of 1G, 2G, and 3G ONTs.

Material	Level of Adhesion
Ti-13Nb-13Zr	High
1G ONTs	High
2G ONTs	Medium
3G ONTs	Medium

**Table 6:** Number of samples assigned to each expert.

	Expert 2					
**Expert 1**		Class 1	Class 2	Class 3	Class 4	Class 5
	Class 1	5	0	0	0	0
	Class 2	1	4	0	0	0
	Class 3	0	1	8	1	0
	Class 4	0	0	1	9	1
	Class 5	0	0	0	0	7

**Table 7:** Results of the post-hoc test.

	1G ONTs	2G ONTs	3G ONTs	Ti-13Nb-13Zr
1G ONTs		0.0030	0.1520	0.5908
2G ONTs	0.0030		0.0000	0.4582
3G ONTs	0.1520	0.0000		0.0017
Ti-13Nb-13Zr	0.5908	0.4582	0.0017	

## Data Availability

MDPI Research Data Policies.
